# Family hardiness and associated factors among Chinese family caregivers of people with Alzheimer’s disease: an explanatory sequential mixed-methods study

**DOI:** 10.3389/fpsyg.2026.1940356

**Published:** 2026-10-14

**Authors:** Xuefen Lan, Minqi Xia, Lihong Chen, Simin Zheng, Lihong Jin, Yini Wang, Shunfei Lu, Jiamin Xu, Linyan Xu, Caifu Li, Ping Yan

**Affiliations:** 1 Faculty of Nursing, College of Medicine, Lishui University, Lishui, China; 2 Department of Nursing, The First Affiliated Hospital of Lishui University, Lishui People’s Hospital, Lishui, China

**Keywords:** Alzheimer’s disease, associated factors, family caregivers, family hardiness, mixed methods

## Abstract

**Aim:**

To examine family hardiness and associated factors among Chinese family caregivers of people with Alzheimer’s disease (AD), and to explore caregivers’ experiences of family adaptation in daily care.

**Methods:**

An explanatory sequential mixed-methods design was used. In the quantitative phase, 201 family caregivers recruited from a tertiary hospital in Zhejiang Province completed the Family Hardiness Index (FHI) and measures of potential associated factors. Multivariable linear regression examined associations with total and subscale FHI scores. Twelve caregivers subsequently took part in semi-structured interviews. Directed content analysis was used to interpret the quantitative findings in context and identify additional themes. The two phases were integrated through a following-a-thread approach and a convergence coding matrix.

**Results:**

The mean total FHI score was 56.85 ± 6.27. Commitment/responsibility had the highest mean subscale score (26.09 ± 3.71), and challenge the lowest (13.49 ± 2.04). In the final model for total FHI score (adjusted R^2^ = 0.477), family resources (β = 0.390, *p* < 0.001), female patient sex (β = -0.286, *p* = 0.005), perceived caregiving burden (β = -0.262, *p* < 0.001), and family meaning-making (β = 0.190, *p* = 0.003) were independently associated with family hardiness. The qualitative analysis generated eight themes. Integration yielded two convergent findings, five complementary findings, and one exploratory theme found only in the interviews: companion-animal support.

**Conclusion:**

Family resources, perceived caregiving burden, family meaning-making, and patient sex were associated with total FHI scores. Interviews provided context on family obligation, changing roles, and urban-rural differences in access to support. Companion-animal support, mentioned by two participants, is reported as a hypothesis-generating observation. Prospective studies are needed to examine causal relationships and the effects of family-support interventions.

## 1 Introduction

Alzheimer’s disease (AD) is a progressive neurodegenerative disorder of the central nervous system, characterized by cognitive decline and behavioral disturbances, with advancing age as the primary risk factor (Tenchov et al., 2024). As the leading cause of dementia, AD accounts for 60%–70% of cases worldwide (Xu et al., 2023). Its global burden is rapidly increasing, with the World Health Organization projecting cases to rise from 55 million in 2019 to 139 million by 2050 (World Health Organization [WHO], 2021). China bears a disproportionate share, with prevalence expected to climb from 16.99 million (29.8% of the global total) to 30.03 million, positioning the country at the epicenter of this crisis (World Health Organization [WHO], 2021). AD has emerged as the third leading threat to older adults’ health, surpassed only by cardiovascular disease and cancer (World Health Organization [WHO], 2021).

The disease’s insidious onset and irreversible progression result in profound cognitive and functional impairments, often accompanied by neuropsychiatric symptoms, necessitating prolonged, specialized care (Porsteinsson et al., 2021). In China, approximately 70% of families provide care independently (Yun and Xiao, 2015), placing substantial psychological, physical, and economic burdens on caregivers. Over half report anxiety or depression and declining health (Wang et al., 2022), while employment disruptions exacerbate financial strain (Zhou and Wang, 2015). Regional disparities are evident; for example, Zhejiang Province exhibits a higher prevalence (10.34%) than many other regions (Pan et al., 2022).

The effects of AD extend beyond cognitive impairment. Healthy ageing is defined in terms of the functional ability that enables well-being in later life (World Health Organization [WHO], 2015). As AD progresses, autonomy and social participation may diminish, while sustained caregiving may affect caregivers’ physical and psychological health (Parra-Rizo and Sanchis-Soler, 2022). Family-oriented physical activity programmes have shown potential benefits for patient functioning and perceived caregiver burden, although findings vary by programme and setting (Sanchís-Soler et al., 2026). Family adaptation should therefore be considered alongside the functioning and well-being of both care recipients and caregivers.

Family resilience is the broader, context-dependent process through which families mobilize resources, construct meaning, reorganize relationships, and adapt to sustained adversity (Abulaiti et al., 2022; Chu et al., 2022; Li and Wang, 2018; Santonja-Ayuso et al., 2024). Family hardiness is related but narrower: it refers to an internal family strength characterized by commitment, perceived control, and viewing change as a challenge. In this study, family resilience provides the theoretical framework, whereas family hardiness is the construct measured quantitatively with the FHI. The qualitative phase addresses caregivers’ experiences of family adaptation and is used to contextualize, not extend, the construct measured by the FHI.

Most family-resilience research has been conducted in Western settings, while Chinese evidence remains comparatively limited and has focused mainly on cancer, stroke, or dementia in general (Chu et al., 2022; Wang, 2023; Xu, 2024; Zhang et al., 2023). Because caregiving is embedded in family relationships and social expectations, models developed elsewhere may require cultural interpretation when applied in China. Filial responsibility, intergenerational reciprocity, and family hierarchy may be relevant, but their meaning and influence are not uniform across Chinese families.

McCubbin’s Family Adjustment and Adaptation Response (FAAR) model provides a framework for examining how demands, resources, appraisals, and coping relate to family adaptation (Nichols, 2013). Its application in China requires attention to local family structures, values, and caregiving expectations (Duangjina et al., 2025; Wang, 2018). Wang (2018) adapted this framework by incorporating cognitive appraisal, contextual meaning-making, and family paradigms. On this basis, the present study considered seven domains: patient-related factors, caregiver characteristics, family resources, stressors, cultural cognition, support systems, and coping strategies (Bang and Kim, 2016; Chu et al., 2022; Deist and Greeff, 2014; Donnellan et al., 2017; Duangjina et al., 2025; Fitryasari et al., 2018; Gur et al., 2024; Hwang et al., 2018; Mao et al., 2021; Qiao and Tang, 2021; Tang et al., 2023; Wang, 2018; Xin, 2022; Zhang et al., 2023, 2025).

Three limitations in the Chinese literature informed this study. First, many studies lack an explicit family-level framework, which limits comparison across findings. Second, culturally adapted models have not always been examined across their full range of domains, particularly family paradigms and caregiving roles. Third, cross-sectional surveys predominate, leaving limited evidence on how caregivers describe family adaptation and the caregiving experiences surrounding measured family hardiness.

We therefore used an explanatory sequential mixed-methods design. The quantitative phase examined family hardiness and its associations with factors representing Wang’s (2018) model. The qualitative phase explored caregivers’ experiences of family adaptation in daily care and allowed additional sources of support to emerge. Integration was used to place the statistical findings within their caregiving and sociocultural context.

The study addressed the following research questions:

(1) What are the levels of family hardiness and its control, commitment/responsibility, and challenge dimensions among Chinese family caregivers of people with AD?

(2) Which factors representing the domains of Wang’s (2018) Chinese Family Resilience Model are independently associated with overall family hardiness and its dimensions?

(3) How do family caregivers describe family adaptation and the factors examined quantitatively in the context of daily AD care, and what additional sources of support emerge from their accounts?

## 2 Materials and methods

### 2.1 Study design

An explanatory sequential mixed-methods design (QUAN → qual) was used (Creswell and Plano Clark, 2011). A cross-sectional survey first examined factors associated with family hardiness. Caregivers with varied FHI scores and sociodemographic characteristics were then purposively selected for semi-structured interviews on family adaptation and related experiences in daily care. Directed content analysis combined framework-based categories with inductive coding (Assarroudi et al., 2018). Integration occurred through participant selection, development of the interview guide from the quantitative findings, and comparison of both datasets in a joint display. The Institutional Review Board of Lishui People’s Hospital approved the study (Approval No. 2025015-01).

### 2.2 Participants and setting

The study was conducted in the neurology outpatient department of a tertiary hospital in Lishui, Zhejiang Province, China. The department includes a specialist memory clinic and serves both urban and rural populations in Lishui and surrounding areas.

Participants were recruited between May and August 2025 using convenience sampling of family caregivers of AD patients attending the clinic. Inclusion criteria: (1) Patients were required to have a confirmed diagnosis of AD according to the 2018 Chinese Guidelines for the Diagnosis and Treatment of Dementia and Cognitive Impairment (II): Guidelines for the Diagnosis and Treatment of Alzheimer’s Disease (Writing Group of the Chinese Guidelines for the Diagnosis and Treatment of Dementia and Cognitive Impairment and Cognitive Disorders Committee of the Neurologists Branch of the Chinese Medical Doctor Association, 2018); (2) caregivers aged ≥ 18 years; (3) family members (spouse, adult child, or cohabiting relative); (4) primary caregivers for ≥ 6 months providing ≥ 4 h/day of care (Zhang, 2023); (5) intact cognitive and communicative abilities; and (6) able to provide informed consent. Exclusion criteria: caregivers of institutionalized patients or professionally compensated caregivers.

The quantitative phase assessed 26 independent variables. Using a conventional allowance of 5–10 participants per variable for multivariable analysis (Ni et al., 2010), and allowing for 20% invalid or incomplete questionnaires, the target sample was 163–325. Of 210 questionnaires distributed, 201 were valid, giving a response rate of 95.7%.

For the qualitative phase, purposive sampling sought variation in FHI scores, caregiver-patient relationship, urban or rural residence, and sociodemographic background.

### 2.3 Procedure

Following IRB approval, researchers collaborated with outpatient staff to identify eligible caregivers. Data collection followed a standardized three-stage protocol:

Informed consent: Researchers explained study objectives, procedures, and participants’ rights, including voluntary participation and withdrawal. Written consent was obtained prior to participation. Multimodal survey administration: Questionnaires were completed (a) on-site with assistance for older or low-literacy participants, (b) take-home with WeChat-based follow-up and photo verification within 1 week, or (c) via structured telephone interviews for mobility-limited caregivers. Researchers were available for clarification, and reminder procedures were implemented.

Questionnaire completion took approximately 30–50 min. Responses were checked for completeness and data entry was independently verified. Missing values comprised 1.39% of all data fields and occurred mainly in the general social-support and community-support items. Where possible, incomplete or ambiguous responses were clarified with participants and recorded as verified source data. Two questionnaires with more than 20% missing responses were excluded. Among the retained questionnaires, unresolved missing demographic or clinical values were replaced with the mean for the relevant variable, and missing scale items with the item median. Multiple imputation was not used.

The interview guide was developed after the quantitative analysis. Survey participants were purposively invited and provided separate written consent for the interview. Interviews took place in a private room at the hospital or in participants’ homes. Data collection and preliminary analysis proceeded concurrently. After interview 11 yielded no new codes, one further interview was conducted; it did not materially change the coding framework, and recruitment ended.

### 2.4 Quantitative phase

#### 2.4.1 Data collection

A structured sociodemographic and clinical questionnaire was developed with reference to previous studies and Wang’s (2018) family resilience framework (Chu et al., 2022; Wang, 2018). Patient-related information included sex, age, education, residence, disease duration, and disease severity. Caregiver-related information included sex, age, relationship to the patient, education, self-rated health, employment status, number of children, and monthly household income.

Family hardiness was assessed using the Family Hardiness Index (FHI), developed by McCubbin et al. (1986) within the FAAR framework (McCubbin and Patterson, 1983). The Chinese version was evaluated by Liu et al. (2014). This 20-item caregiver-reported instrument comprises three dimensions: control (items 1–3, 10, 19, and 20), commitment/responsibility (items 4–9, 11, 13, and 18), and challenge (items 12 and 14–17). Items are rated on a four-point Likert scale from 1 (strongly disagree) to 4 (strongly agree), giving a total score of 20–80; higher scores indicate greater family hardiness. Cronbach’s alpha was 0.803 in the Chinese evaluation and 0.790 in this sample. Within McCubbin’s resiliency model of family stress, coping, and adaptation, family hardiness is an internal family strength rather than a synonym for family resilience (McCubbin et al., 1996). Accordingly, the FHI was used to measure family hardiness as one indicator situated within the broader family resilience framework. All FHI-based quantitative findings are therefore reported as family hardiness, not family resilience.

Guided by Wang’s (2018) model and previous research (Chu et al., 2022), candidate variables were organized into seven broad domains: patient-related factors, caregiver characteristics, family resources, stressors, cultural cognition, support systems, and problem-solving and coping. Patient-related factors included functional impairment and behavioral symptoms. Functional impairment was assessed using the 20-item Chinese Lawton–Brody Activities of Daily Living Scale (ADL-C). Scores range from 20 to 80, with higher scores indicating greater functional impairment. Cronbach’s alpha was 0.983 in the present sample. Behavioral symptoms were assessed using the 24-item Revised Memory and Behavior Problems Checklist (RMBPC). Items were scored as absent (0) or present (1), producing a total score from 0 to 24; higher scores indicate more behavioral problems. Cronbach’s alpha was 0.900 in the present sample.

Perceived caregiving burden was assessed using the 22-item Chinese version of the Zarit Burden Interview (ZBI-C; Wang et al., 2006). Items are rated on a five-point scale from 0 (never) to 4 (nearly always), yielding a total score from 0 to 88. Higher scores indicate greater perceived caregiving burden. Cronbach’s alpha was 0.946 in the present sample.

Family coherence was assessed using the three-item Chinese Family Coping Coherence Index (FCCI-C). Items are rated on a five-point scale, producing a total score from 3 to 15; higher scores indicate a more positive and coherent family appraisal of stressful circumstances. Cronbach’s alpha was 0.692 in the present sample. Family schema was measured using the 30-item Chinese Family Schema–Ethnic Index (FSCH-E-C). Substantive responses are rated from 0 to 3, and the option “never considered” is coded -1. Total scores range from -30 to 90, with higher scores indicating stronger endorsement of the family beliefs and values represented by the scale. Cronbach’s alpha was 0.898 in the present sample.

Family paradigms were assessed using the 33-item Family Paradigms Scale developed by Wang (2018). Items are rated from 0 (not considered) to 4 (consistently present), yielding a total score from 0 to 132. Higher scores indicate stronger endorsement of the family norms and caregiving patterns assessed by the instrument. Cronbach’s alpha was 0.838 in the present sample. Family meaning-making was assessed using the nine-item Chinese Positive Aspects of Caregiving Scale. The scale comprises self-affirmation (items 1–4) and outlook on life (items 5–9). Items are rated on a five-point Likert scale from 1 (strongly disagree) to 5 (strongly agree), producing a total score from 9 to 45. The response options “do not know” (coded -3) and “refuse to answer” (coded -4) represent non-substantive responses and were treated as missing data rather than included in the total score. Higher scores indicate a more positive appraisal of the caregiving experience. Cronbach’s alpha was 0.903 in the present sample.

Family resources were measured using the 51-item Chinese Family Inventory of Resources for Management (FIRM-C). The instrument covers family control and health status, economic capacity, and dignity and communication. Items are rated from 0 (never) to 3 (always), yielding a total score from 0 to 153. Higher scores indicate greater perceived family resources. Cronbach’s alpha was 0.927 in the present sample. Support systems were assessed using four measures. Perceived family support was measured using a four-item subscale scored from 4 to 28 (α = 0.882); kinship support was measured using four items scored from 0 to 12 (α = 0.613); community support was assessed using eight items scored from 8 to 56 (α = 0.933); and general social support was assessed using six items scored from 6 to 42 (α = 0.899). Higher scores on each measure indicate greater perceived support.

Problem-solving and coping were assessed using the 27-item Chinese Family Crisis-Oriented Personal Evaluation Scales (F-COPES-C). The instrument covers resource acquisition, help-seeking, positive reframing, spiritual coping, and avoidance. Items are rated on a five-point Likert scale, producing a total score from 27 to 135; higher scores indicate greater use of adaptive coping strategies after scoring applicable items according to the instrument instructions. Cronbach’s alpha was 0.843 in the present sample. Further information on the response formats, subscale composition, Chinese-language sources, and available psychometric evidence for each instrument is provided in Supplementary Table 1.

### 2.5 Qualitative phase

#### 2.5.1 Qualitative data collection

Individual semi-structured, in-depth interviews were conducted to explore caregivers’ experiences and explore caregivers’ experiences of family adaptation and their perceived sources of support and difficulty in AD caregiving. The interview guide was developed through a culturally grounded, multi-step process: (1) alignment with China’s Family Resilience Model (Wang, 2018); (2) integration of quantitative findings; and (3) iterative refinement via an expert panel of three gerontology specialists. Pilot testing with three primary caregivers (not included in final data analysis) informed revisions enhancing cultural relevance, conceptual clarity, and clinical sensitivity. Key questions included: “How did your family first notice the patient’s symptoms?” “What were your initial coping responses?” “Which resources supported you during crises?” and “How has caregiving reshaped your family’s outlook?”

All interviews were conducted by the corresponding author (PY), a nurse-researcher with geriatric clinical experience at the study hospital. She had previous professional contact with some participants. This familiarity may have supported rapport, but it may also have shaped disclosure. Interviews lasted 30–60 min, were audio-recorded with consent, transcribed verbatim, and de-identified. Reflexive field notes recorded the interview context, emerging interpretations, and researcher-participant interactions and were considered during analysis (Bott, 2010).

#### 2.5.2 Qualitative data analysis

Directed content analysis followed Hsieh and Shannon (2005) and Assarroudi et al. (2018). Initial categories were derived from the family resilience framework: patient characteristics, family coherence, family paradigms, family meaning-making, responses to caregiving demands, family resources, and problem-solving and coping. Material outside these categories was coded inductively, leading to themes including reconfigured family roles and companion-animal support.

#### 2.5.3 Trustworthiness of qualitative study

Trustworthiness was addressed using Lincoln and Guba’s (1985) criteria. Two researchers independently coded all 12 transcripts and resolved differences through discussion and review of the source text; a third researcher was consulted when needed. Field notes were used to examine the influence of context and researcher position on interpretation. Three participants commented on preliminary thematic summaries, and the team used peer debriefing. The decision to stop recruitment after 12 interviews reflected the absence of new codes in interviews 11 and 12, the focused aim, and the information power of the sample (Malterud et al., 2016).

### 2.6 Data integration

Quantitative and qualitative findings were integrated using a “following-a-thread” approach and a convergence coding matrix (O’Cathain et al., 2010). Quantitative screening (*n* = 201) identified family hardiness scores, guiding purposive qualitative sampling (*n* = 12) across diverse demographics. Results from regression analyses and content analysis were mapped in the matrix as convergent, complementary, dissonant, or silent. Discrepancies were resolved via expert panel review. This process adhered to rigorous mixed-methods standards (Creswell and Plano Clark, 2011).

## 3 Results

### 3.1 Quantitative phase

#### 3.1.1 Participant characteristics

The study recruited 210 participants, excluding 7 who declined for busy caregiving and 2 with invalid questionnaires (>20% missing data). As shown in Table 1, the 201 patients with AD were evenly distributed by sex (49.8% male, 50.2% female). Most had low educational attainment (63.7% primary school or less; 3.9% college graduates), and rural residents slightly outnumbered urban counterparts (53.2% vs. 46.8%). Disease duration varied, with 35.3% of patients diagnosed for 5–10 years and 31.8% newly diagnosed ( < 3 years). Severe dementia (31.8%) and questionable dementia (28.4%) were the most common.

**TABLE 1:** Demographic and clinical characteristics of people with AD (*N* = 201).

Characteristics	*n* (%)
**Age**	
41∼59	9 (4.5)
≥ 60	192 (95.5)
**Gender**	
Male	100 (49.8)
Female	101 (50.2)
**Education Level**	
Primary school or below	128 (63.7)
Junior high school	52 (25.9)
Senior high/vocational	13 (6.5)
College or above	8 (3.9)
**Residence**	
Urban	94 (46.8)
Rural	107 (53.2)
**Disease duration (years)**	
< 3	64 (31.8)
3–5	41 (20.4)
6–10	71 (35.3)
> 10	25 (12.5)
**Disease severity**	
Questionable dementia	57 (28.4)
Mild dementia	49 (24.4)
Moderate dementia	31 (15.4)
Severe dementia	64 (31.8)

These data describe the care recipients. AD, Alzheimer’s disease.

Among caregivers, 53.7% were aged ≥ 60 years and 62.7% were female. Kinship distribution indicated that adult children (51.2%) and spouses (37.3%) were the primary caregivers. Educational levels were modest (41.3% with elementary school), though 67.7% reported good health. Household income was moderate for most families (52.7%, 3,000–6,000 RMB/month), and the median number of children per household was 2 (IQR: 2–3; Supplementary Table 2).

#### 3.1.2 Family hardiness scores

As shown in Table 2, Family Hardiness Index scores ranged from 43 to 77 (mean 56.85 ± 6.27). The commitment/responsibility dimension had the highest mean score (26.09 ± 3.71), followed by control (17.27 ± 2.39) and challenge (13.49 ± 2.04).

**TABLE 2:** Family Hardiness Index scores among family caregivers of people with AD (*N* = 201).

Dimensions	Min	Max	M ± SD
Total	43	77	56.85 ± 6.27
Control	8	23	17.27 ± 2.39
Commitment/ responsibility	12	35	26.09 ± 3.71
Challenge	7	20	13.49 ± 2.04

AD, Alzheimer’s disease.

#### 3.1.3 Factors associated with family hardiness among family caregivers of people with AD

Multivariable linear regression was conducted for the total FHI score and each subscale. Variables associated with the relevant outcome at *p* < 0.05 in univariable analyses were entered as candidates; categorical coding is reported in Supplementary Table 3. The final total-score model explained 47.7% of adjusted variance (*F* = 46.60, *p* < 0.001). Higher family resources (β = 0.390, *p* < 0.001) and meaning-making scores (β = 0.190, *p* = 0.003) were associated with higher FHI scores, whereas female patient sex (β = −0.286, *p* = 0.005) and greater caregiving burden (β = −0.262, *p* < 0.001) were associated with lower scores (Table 3). Rural residence and caregiving burden were negatively associated with control, while meaning-making was positively associated with it (adjusted R^2^ = 0.256). Commitment/responsibility was positively associated with problem-solving and coping, family paradigms, and meaning-making, and negatively associated with female patient sex and caregiving burden (adjusted R^2^ = 0.402). Challenge was positively associated with family resources and negatively associated with caregiving burden (adjusted R^2^ = 0.336; Supplementary Tables 4–6).

**TABLE 3:** Multivariable linear regression model for total Family Hardiness Index score among family caregivers of people With AD (*N* = 201).

Variables	Standardized β	*p*
Family resources	0.390	< 0.001
Patient sex (female vs. male)	−0.286	0.005
Perceived caregiving burden	−0.262	< 0.001
Family meaning-making	0.190	0.003

R^2^ = 0.487; adjusted R^2^ = 0.477; *F* = 46.60, *p* < 0.001; Durbin–Watson = 2.006; maximum VIF = 1.77. AD, Alzheimer’s disease; VIF, variance inflation factor.

### 3.2 Qualitative phase

#### 3.2.1 Interviewee characteristics

The qualitative phase included semi-structured interviews with 12 family caregivers of outpatient patients with AD. Caregivers ranged in age from 41 to 90 years and included seven males and five females. Educational levels varied: two participants had primary education or below; two, junior high school; three, senior high school or vocational training; and five, college education or higher. Nine care recipients resided in urban areas and three in rural areas. Caregiver-patient relationships included spouses (*n* = 5), sons (*n* = 5), and daughters-in-law (*n* = 2). Caregiving duration ranged from less than 2 years (*n* = 5) to 3–5 years (*n* = 4) and over 5 years (*n* = 3). Participants’ occupations included self-employment (*n* = 5), retirement (*n* = 4), and unemployment (*n* = 3). Detailed characteristics are presented in Supplementary Table 7.

#### 3.2.2 Interview findings

Directed content analysis of the 12 interviews produced eight themes and 17 subthemes (Table 4). The family resilience framework guided the initial categories, while material that did not fit them was retained through inductive coding. Companion-animal support emerged inductively. Quotations are identified by participant code.

**TABLE 4:** Qualitative themes and representative quotations from family caregivers of people with AD (*N* = 12).

Theme	Sub-theme	Representative quotation(s)
Family role reconstruction under disease impact	Disruption of established family roles	“My wife developed this illness at an unusually early age… The symptoms began around her retirement. It is still hard to accept an early-onset diagnosis… Now she has severe functional impairment and needs almost complete assistance with daily activities, which places a heavy burden on caregiving.” (P4, Husband)
	Role transition and role reversal	“I used to focus on work and school matters, while she managed the household—our child’s education, daily chores… She was cheerful and active, and our home was always lively. Now I’m the one caring for her. At the beginning, I knew nothing about caregiving… When someone becomes ill like this, there’s no one left to talk to.” (P4, Husband)
Resource constraints in rural settings	Limited access to medical information and professional support	“Community doctors rarely come, maybe once a year, to check blood pressure or blood sugar for the whole village. They don’t deal with conditions like Alzheimer’s at all.” (P7, Wife)
	Limited informal support services	“Hiring a professional caregiver isn’t realistic. Village eldercare services are unregulated, and welfare applications take too long, so I care for her myself… My mother sleeps beside me, and I’ve set up a small cot nearby. Since her illness began, I’ve been sleeping about 4 h a day.” (P6, Son)
Family meanings of caregiving situations	Self-actualization through caregiving	“Now it’s about saving our parents—the longer we can keep them going, the more it proves our worth as children.” (P1, Son)
	Enhanced family cohesion	“After she became ill, my relationship with my younger brother actually improved… He said money isn’t the issue, even if we need to borrow, we’ll make sure she gets proper care.” (P6, Son)
Family paradigms in Alzheimer’s caregiving	Filial piety values	“In our family, everyone values filial piety. When the elder was hospitalized, all five sisters took turns caring for him, which made the pressure manageable (laughs).” (P5, Daughter-in-law)
	Equal, open, and mutually respectful communication	“About a year and a half ago, we realized she would forget to turn off the stove… After discussing it with my siblings, we decided that the four of us would take turns bringing her home for a month at a time. She doesn’t want to go to a nursing home because of language barriers, so for now, we’ll continue this arrangement and later discuss hiring a live-in caregiver or institutional care.” (P2, Son)
Responses to changing care demands	Early-stage stress appraisal	“She carries a GPS phone, an ID card in her bag, and a tracking necklace bought by our son, all with her name, my contact details, and real-time location.” (P4, Husband)
	Mid-to-late-stage stress appraisal	“When I discovered a sacral pressure ulcer, I panicked and tried random ointments. Only after a hospital referral did we learn about wound-care specialists, whose month-long home visits resolved the problem.” (P4, Husband)
Family resources	Sense of dignity among family members	“We couldn’t force her to move to her younger son’s home. We have to respect her wishes.” (P5, Daughter-in-law)
	Health status of family members	“I have hypertension, high blood sugar, and swollen feet… I asked my husband, ‘Why is life so hard on me?’ (crying). Everything falls on me, I just get through each day.” (P5, Daughter-in-law)
	Family financial situation	“He retired early and receives over 8,000 yuan a month in pension. Hospitalizations and hiring caregivers aren’t a problem, and most expenses are reimbursed.” (P11, Wife)
Problem-solving and coping strategies	Responsibility allocation	“Her sons and daughters-in-law take turns with cooking, laundry, and daily care.” (P9, Daughter-in-law)
	Acceptance of reality	“Recovery is impossible. What else can we do? I’ve stopped dwelling on it.” (P8, Wife)
	Self-regulation	“I keep up with hobbies like blogging and photography. Driving to the lake at dusk to take photos helps me unwind.” (P4, Husband)
Companion-animal support	Companion-animal support	“Our 13-year-old cat stays on Mom’s bed, which softens my temper. My retired sister also interacts with pets to avoid isolation.” (P1, Son) “Our dog helps me release stress. I talk to it and jog along the floodwall with it. The benefits far outweigh the minor effort.” (P12, Son)

Quotations are identified by participant code and relationship to the care recipient. AD, Alzheimer’s disease.

##### 3.2.2.1 Family role reconstruction under disease impact

Caregivers described changes in household and relational roles when a family member who had previously carried substantial responsibilities became dependent on care. A few also spoke of early onset or rapid decline in the person they cared for; these statements are reported as individual experiences, not evidence of sex differences in AD progression.

###### 3.2.2.1.1 Disruption of established family roles

Increasing dependency altered household responsibilities and added practical and emotional demands. The accounts below describe individual family experiences and were not used to infer differences in disease course by patient sex.

*“My wife developed this illness at an unusually early age… The symptoms began around her retirement. It is still hard to accept an early-onset diagnosis… Now she has severe functional impairment and needs almost complete assistance with daily activities, which places a heavy burden on caregiving.” (P4, Husband)*

*“She has been ill for six years, with no clear hope of recovery. She remains persistently depressed and still relies on a feeding tube… Caring for her requires six tube feedings a day. If this continues for another year or two, both my mother and I may reach our limits.” (P10, Son)*

###### 3.2.2.1.2 Role transition and role reversal

Several participants described wives or mothers as having previously taken primary responsibility for household organization and family relationships. When these women became dependent on care, other family members assumed unfamiliar roles. Caregivers spoke of feeling unprepared, stressed, or isolated during this transition. These accounts reflect individual family experiences and should not be generalized to all families caring for women with AD.

*“I used to focus on work and school matters, while she managed the household-our child’s education, daily chores… She was cheerful and active, and our home was always lively. Now I’m the one caring for her. At the beginning, I knew nothing about caregiving… When someone becomes ill like this, there’s no one left to talk to.” (P4, Husband)*

##### 3.2.2.2 Resource constraints in rural settings

Rural participants reported limited access to professional services and AD-specific caregiving guidance. Some felt insufficiently prepared for the demands of care. As the interviews were not designed for systematic urban–rural comparison, these accounts should be interpreted within the study sample.

###### 3.2.2.2.1 Limited access to medical information and professional support

Some urban participants had received medical information, home-based nursing care, or caregiver education. They considered these services helpful in understanding AD and managing specific care tasks. However, the study did not assess their effects on caregiving competence, anxiety, family hardiness, or family resilience.

*“It was difficult for me to push the wheelchair to the hospital, but a wound care nurse came to our home instead… She changed the dressing every two days for over a month and taught me how to reposition him every two hours. She even suggested buying special cushions online. I had no idea about any of this before.” (P4, Husband)*

In contrast, rural caregivers described minimal engagement from community healthcare services, particularly for dementia-related care.

*“Community doctors rarely come, maybe once a year, to check blood pressure or blood sugar for the whole village. They don’t deal with conditions like Alzheimer’s at all.” (P7, Wife)*

###### 3.2.2.2.2 Limited informal support services

Some participants regarded paid domestic or caregiving assistance as a source of respite. Rural caregivers more often described relying on unpaid family care because formal support was unavailable or unaffordable. Their accounts included sleep loss, physical exhaustion, and difficulty managing continuous care. Burnout was not assessed.

*“Hiring a professional caregiver isn’t realistic. Village eldercare services are unregulated, and welfare applications take too long, so I care for her myself… My mother sleeps beside me, and I’ve set up a small cot nearby. Since her illness began, I’ve been sleeping about four hours a day.” (P6, Son)*

*“We can’t afford paid help… With no support, we manage on our own; it’s exhausting. Nursing homes aren’t an option either… I’ve read online about caregivers mistreating patients, which makes us even more hesitant.” (P7, Wife)*

##### 3.2.2.3 Family meanings of caregiving situations

Participants described finding meaning in caregiving through a sense of personal value and closer family relationships. Some related these experiences to filial responsibility and intergenerational reciprocity. The findings do not establish that these interpretations reduced burnout or sustained long-term family adaptation.

###### 3.2.2.3.1 Self-actualization through caregiving

Some caregivers regarded the continued survival and well-being of the person with AD as evidence that their care was meaningful. This interpretation gave caregiving a sense of purpose. Whether meaning-making affected self-efficacy or the family’s capacity to sustain care was not examined.

*“Now it’s about saving our parents-the longer we can keep them going, the more it proves our worth as children.” (P1, Son)*

*“It’s not suffering. Caring for them for 10, 20, even 30 years would be ideal… The longer they live, the more meaningful our care becomes. We push ourselves like robots (laughs).” (P6, Son)*

###### 3.2.2.3.2 Enhanced family cohesion

Several caregivers reported closer relationships, shared priorities, and greater mutual support as care needs increased. Responsibility was often distributed among family members, and caregiving became a shared family concern.

*“Everyone is willing to care for Mom, and no one sees it as a burden… My three daughters come every day and patiently look after her. It has brought us closer.” (P3, Husband)*

*“After she became ill, my relationship with my younger brother actually improved… He said money isn’t the issue, even if we need to borrow, we’ll make sure she gets proper care.” (P6, Son)*

##### 3.2.2.4 Family paradigm in Alzheimer’s caregiving

Participants discussed two aspects of family paradigms: filial obligation and communication about caregiving roles. Some viewed filial responsibility as a moral basis for shared care, whereas others emphasized discussion and negotiation when allocating time, tasks, and financial responsibilities.

###### 3.2.2.4.1 Filial piety values

Some participants described filial piety as a moral basis for caring for an older relative. In these families, a shared sense of obligation accompanied rotational care or division of tasks. The findings do not show that filial values reduced caregiver burden or operated uniformly across families.

*“In our family, everyone values filial piety. When the elder was hospitalized, all five sisters took turns caring for him, which made the pressure manageable (laughs).” (P5, Daughter-in-law)*

*“Every family or clan has its own ethos. Filial piety should be a tradition, caring for elders should feel natural and expected.” (P12, Son)*

###### 3.2.2.4.2 Equal, open, and mutually respectful communication

Participants described open and respectful discussion when allocating caregiving tasks, financial contributions, and future care arrangements. They regarded such communication as helpful in coordinating family decisions.

*“About a year and a half ago, we realized she would forget to turn off the stove… After discussing it with my siblings, we decided that the four of us would take turns bringing her home for a month at a time. She doesn’t want to go to a nursing home because of language barriers, so for now, we’ll continue this arrangement and later discuss hiring a live-in caregiver or institutional care.” (P2, Son)*

*“After talking with my aunt (daughter of the patient) from the same village, she agreed to help. I used to sleep beside her every night, but now we take turns. One of us stays with her at night, and during the day, my aunt sometimes helps bathe her.” (P5, Daughter-in-law)*

##### 3.2.2.5 Responses to changing care demands

Caregivers’ concerns and responses differed according to the care needs they described. Accounts of earlier stages emphasized wandering and safety; those concerning later stages focused on dependency and complications requiring skilled care. These retrospective accounts do not constitute longitudinal evidence of adaptation.

###### 3.2.2.5.1 Early-stage stress appraisal

In accounts of earlier-stage AD, wandering and the risk of becoming lost were common concerns. Caregivers used GPS-enabled devices, identification cards, and location-tracking tools to monitor safety.

*“When she wanders off, it’s almost impossible for her to find her way back. Now we use GPS tracking on her phone. Before that, I had to video-call her and ask bystanders to share her location.” (P2, Son)*

*“She carries a GPS phone, an ID card in her bag, and a tracking necklace bought by our son, all with her name, my contact details, and real-time location.” (P4, Husband)*

###### 3.2.2.5.2 Mid-to-late stage stress appraisal

Accounts of more advanced care needs concerned dysphagia, loss of independence, pressure injuries, fractures, and the accompanying practical and emotional strain. Some caregivers sought assistance from live-in carers with relevant experience or specialist wound-care services. Although participants considered this support useful, its effectiveness was not evaluated.

*“She can no longer eat by mouth and depends on nasogastric feeding. We lack the technical skills, but our live-in caregiver can manage it safely because of her professional training.” (P3, Husband)*

*“When I discovered a sacral pressure ulcer, I panicked and tried random ointments. Only after a hospital referral did we learn about wound-care specialists, whose month-long home visits resolved the problem.” (P4, Husband)*

##### 3.2.2.6 Family resources

Participants identified three resources as particularly relevant to daily care: respect for the care recipient’s dignity, caregiver health, and family financial capacity.

###### 3.2.2.6.1 Sense of dignity among family members

Participants discussed dignity in relation to respecting the care recipient’s preferences and managing anticipated reactions to the diagnosis. Some accounts reflected tension between autonomy and safety. Others described withholding the diagnosis outside the family because of concern about gossip or stigma, which they felt limited opportunities to seek support.

*“We couldn’t force her to move to her younger son’s home. We have to respect her wishes.” (P5, Daughter-in-law)*

*“I never talk about my wife’s condition with classmates or friends. There’s no point; it would only turn into gossip. So, I keep it to myself; only my family knows.” (P4, Husband)*

###### 3.2.2.6.2 Health status of family members

Caregivers with health problems reported difficulty sustaining continuous care. Some also spoke of reduced social contact, loneliness, or emotional strain. Anxiety, depression, and family functioning were not assessed using clinical measures.

*“I have hypertension, high blood sugar, and swollen feet… I asked my husband, ‘Why is life so hard on me?’ (crying). Everything falls on me, I just get through each day.” (P5, Daughter-in-law)*

*“A reduced social life is unavoidable. Caring for her comes first; everything else has to wait.” (P3, Husband)*

###### 3.2.2.6.3 Family financial situation

Participants viewed financial capacity as relevant to meeting long-term medical and care costs and obtaining paid assistance. Those with greater financial security reported fewer difficulties paying for services, whereas others described lost income, limited savings, or an inability to employ additional help.

*“He retired early and receives over 8,000 yuan a month in pension. Hospitalizations and hiring caregivers aren’t a problem, and most expenses are reimbursed.” (P11, Wife)*

*“All three of us work as day laborers at the same construction site. We earn about 100,000 yuan a year, but it’s all spent. Saving is impossible.” (P5, Daughter-in-law)*

##### 3.2.2.7 Problem-solving and coping strategies

Caregivers described three approaches to managing daily care demands: allocating responsibilities, accepting disease-related changes, and regulating their emotions and activities. These represent reported caregiving practices rather than strategies shown to improve family hardiness or sustain family resilience.

###### 3.2.2.7.1 Responsibility allocation

Participants described dividing practical tasks and financial responsibilities among family members. Some considered clear role allocation helpful in coordinating care and avoiding the concentration of responsibility in one person.

*“Our daughter and son have divided responsibilities; she handles hospital care, and he covers the medical expenses.” (P7, Wife)*

*“Her sons and daughters-in-law take turns with cooking, laundry, and daily care.” (P9, Daughter-in-law)*

###### 3.2.2.7.2 Acceptance of reality

Some caregivers described accepting the irreversibility of AD and no longer focusing on recovery. They felt that this helped them manage day-to-day care, although anxiety and emotional stability were not formally assessed.

*“It’s incurable. Many people in rural areas have dementia. There’s simply no treatment.” (P5, Daughter-in-law)*

*“Recovery is impossible. What else can we do? I’ve stopped dwelling on it.” (P8, Wife)*

###### 3.2.2.7.3 Self-regulation

Caregivers reported using hobbies, social contact, and periods of rest to manage increasing demands. The study did not examine whether these practices improved caregiver well-being or family hardiness.

*“I keep up with hobbies like blogging and photography. Driving to the lake at dusk to take photos helps me unwind.” (P4, Husband)*

*“I make myself pause, napping when exhausted or watching TV or TikTok to reset. I feel more energized the next day.” (P9, Daughter-in-law)*

##### 3.2.2.8 Companion-animal support

Two of the 12 caregivers spontaneously mentioned their own or a relative’s pet as a source of companionship, calm, or daily routine during caregiving. Because pets were not included in the interview guide or quantitative measures, this finding is treated as exploratory and hypothesis-generating rather than as a dimension of family resilience.

*“Our 13-year-old cat stays on Mom’s bed, which softens my temper. My retired sister also interacts with pets to avoid isolation.” (P1, Son)*

*“Our dog helps me release stress. I talk to it and jog along the floodwall with it. The benefits far outweigh the minor effort.” (P12, Son)*

### 3.3 Integration phase

Table 5 brings together the quantitative associations and qualitative accounts. Family meaning-making and family resources were classified as convergent; patient sex, residence, family paradigms, caregiving burden and responses to care demands, and problem-solving and coping as complementary. Companion-animal support appeared only in the qualitative phase and was classified as exploratory.

**TABLE 5:** Integration of quantitative family hardiness findings and qualitative family adaptation findings.

Integrated topic	Quantitative data	Qualitative data	Integration assessment
Patient sex (female)	Female patient sex was associated with lower total family hardiness (β = −0.286, *p* = 0.005) and commitment/responsibility (β = −0.249, *p* = 0.020).	Caregivers described the shift of a wife or mother into a dependent role as disrupting established family roles and increasing caregiving demands. Some also perceived an earlier or more rapid disease course; this was not tested quantitatively.	Complementary
Patient residence (rural)	Rural residence was associated with lower control scores (β = −0.348, *p* = 0.004).	Rural participants described limited access to dementia-specific information, professional services, respite care, and paid caregiving support.	Complementary
Family meaning-making	Higher scores were associated with higher total family hardiness (β = 0.190, *p* = 0.003), control (β = 0.202, *p* = 0.002), and commitment/responsibility (β = 0.163, *p* = 0.019).	Caregivers described finding purpose in sustained care, filial responsibility, and greater family cohesion.	Convergent
Family paradigms	Higher scores were associated with higher commitment/responsibility (β = 0.220, *p* < 0.001).	Participants described filial obligation, shared roles, and open communication when organizing care.	Complementary
Perceived caregiving burden and responses to changing care demands	Greater perceived burden was associated with lower total family hardiness (β = −0.262, *p* < 0.001), control (β = −0.364, *p* < 0.001), commitment/responsibility (β = −0.236, *p* < 0.001), and challenge (β = −0.179, *p* = 0.004).	Caregivers described safety planning in earlier-stage care and seeking skilled support as dependency and complications increased. These accounts concerned responses to care demands rather than perceived burden itself.	Complementary but conceptually distinct
Family resources	Higher scores were associated with higher total family hardiness (β = 0.390, *p* < 0.001) and challenge (β = 0.475, *p* < 0.001).	Participants identified financial capacity, caregiver health, patient dignity, and access to support as resources relevant to daily care.	Convergent
Problem-solving and coping	Higher scores were associated with higher commitment/responsibility (β = 0.289, *p* < 0.001).	Caregivers described allocating responsibilities, accepting disease-related changes, maintaining hobbies, seeking social contact, and resting when possible.	Complementary
Companion-animal support	N/A	Two of 12 caregivers spontaneously described companion animals as sources of companionship, calm, or daily routine. This theme was not assessed quantitatively.	Qualitative only (exploratory)

Quantitative coefficients are standardized β values from the FHI models. AD, Alzheimer’s disease; FHI, Family Hardiness Index; N/A, not assessed quantitatively.

## 4 Discussion

This study measured family hardiness among Chinese family caregivers of people with AD and used interviews to interpret the quantitative findings within the broader context of family adaptation. Four factors were associated with total FHI score, with additional associations at subscale level. Caregivers discussed family obligation, changing roles, access to services, coping, and resources. Companion-animal support was mentioned by two participants and is treated as exploratory.

### 4.1 Interpreting family hardiness within the family resilience framework

Mean FHI scores differed from those in earlier Chinese studies, although differences in measures, scoring, samples, and settings limit direct comparison (Chu et al., 2022; Tang et al., 2023). Interview participants described shared responsibility, family obligation, and cohesion. Literature on collective coping and familial duty offers one way to interpret these accounts (Tao, 2023), but the present data do not show that cultural values account for the observed scores. The relevance of retirement, multi-child households, and other family arrangements warrants direct examination.

Commitment/responsibility had the highest mean subscale score. Several caregivers described care as a shared obligation and related this responsibility to filial values. This is consistent with literature identifying filial piety as one framework through which some Chinese families understand caregiving (Liu et al., 2022; Wang and Li, 2017). Because filial values were not measured quantitatively, they cannot explain the FHI findings. Their significance is likely to vary by generation, region, family structure, and individual circumstance.

Control and challenge scores were lower than commitment/responsibility scores, but no validated cut-offs were available to classify either domain as impaired. Participants described greater difficulty maintaining control when information, respite care, or financial resources were limited. Disease-specific education and referral to formal services may therefore merit evaluation in future nursing programmes. Participants also recalled changing their responses as care needs evolved, but the cross-sectional design did not examine within-family change or dynamic adaptation (Sun et al., 2023).

### 4.2 Factors associated with family hardiness in AD caregiving

The discussion below considers the quantitative associations with family hardiness alongside caregivers’ accounts of related experiences in daily care.

Female patient sex was associated with lower total FHI and commitment/responsibility scores, in line with Li et al. (2021). Some interviewees described disruption to established family roles after a wife or mother became dependent on care. These accounts show how particular families experienced role change but do not explain the sex association. The study did not compare AD onset, progression, or functional decline between women and men.

Rural residence was associated with lower control scores. Rural participants described limited access to professional services, caregiving information, and respite care, consistent with reported regional inequalities in dementia services (Xin, 2022). This provides context but does not show that limited services caused lower control. Future studies should examine the roles of clinical severity, household responsibilities, and service availability. In practice, nurses should assess these circumstances for each family rather than infer need from residence alone.

More positive family meaning-making was associated with higher total FHI, control, and commitment/responsibility scores, consistent with Opsomer et al. (2020). Caregivers described finding purpose in sustained care and, in some cases, reframing adversity as a challenge. These accounts support consideration of meaning-centered reflection and shared decision-making in future caregiver-support programmes. Directionality remains uncertain: positive meaning-making may support hardiness, while families with greater hardiness may be more able to interpret caregiving positively. Longitudinal research is needed to examine this possible reciprocal relationship.

Family paradigm scores were positively associated with commitment/responsibility, but not with total FHI. Participants discussed filial obligation, shared roles, and open communication when describing how care was organized. These accounts are compatible with literature on family resilience and Confucian-influenced family ethics (Liu, 2024), but they do not demonstrate that filial values or communication patterns increase hardiness. Nursing assessment should elicit each family’s values and preferred division of responsibility rather than assume that filial norms are uniformly shared. Family discussions or educational materials could then be tailored to identified needs and evaluated prospectively.

Greater perceived caregiving burden was associated with lower total and subscale FHI scores. Interviewees described safety planning and seeking professional help as care needs increased. These accounts concern responses to care demands, whereas the ZBI-C measures perceived burden; they are related but not equivalent. Assessment, education, and skills training may be useful components of future stage-sensitive support, but their feasibility and effects were not tested here.

Family resources were positively associated with total FHI and challenge scores. Participants discussed financial capacity, caregiver health, patient preferences, and social recognition as relevant to care. These accounts are consistent with previous family-resilience research, but they do not establish how particular resources influence hardiness (Chu et al., 2022; Deist and Greeff, 2017; Hall et al., 2012).

Some caregivers described financial constraints, health problems, and limited professional support as barriers to sustaining care. Individual assessment may help identify which resources a family lacks. Future research should test whether service access, household finances, and caregiver health account for variation in hardiness and whether resource-oriented support improves outcomes.

Problem-solving and coping were positively associated with commitment/responsibility but not with total FHI. Caregivers described allocating roles, accepting disease-related change, maintaining hobbies, seeking social contact, and resting when possible. These practices resemble coping processes reported in previous family-adaptation research, but their effect on hardiness was not examined (Chu et al., 2022; Hall et al., 2012; Ma, 2021).

The interviews also showed that care concerns differed with the needs being described, from wandering and safety to advanced dependency. Such accounts may help shape future caregiver programmes, although this study did not evaluate stage-specific support. Longitudinal and intervention studies are needed to examine changes in coping and the effects of particular forms of assistance.

Companion-animal support was mentioned spontaneously by two participants, who described companionship, calm, and routine. Neither psychological distress nor effects on family hardiness were measured.

Previous studies have examined companion animals in relation to well-being among dementia caregivers and people with dementia (Connell et al., 2007; Monks and Clark, 2024). The present accounts add a small, context-specific qualitative observation. They do not establish companion-animal support as a dimension of resilience or as a therapeutic intervention. Purpose-designed studies with larger and more diverse samples should examine how often this form of support occurs, when it is helpful or burdensome, and whether it relates to caregiver or family outcomes.

## 5 Limitations

Several limitations should be considered. Recruitment from one tertiary hospital by convenience sampling limits transferability. The cross-sectional analysis cannot establish temporal sequence or causality, and family hardiness was measured at one time point. Missingness was concentrated mainly in the general social-support and community-support items. Item-median imputation for these and other unresolved scale responses may have reduced variability and understated uncertainty. The FHI assesses family hardiness, not the broader and dynamic process of family resilience; the qualitative accounts add context but do not remove this measurement limitation. Internal consistency was below 0.70 for the FCCI-C (α = 0.692) and kinship-support subscale (α = 0.613), increasing the possibility of measurement error. The interviewer’s prior professional contact with some participants may have influenced disclosure. In addition, directing part of the qualitative analysis from statistically significant findings may have reduced attention to other relevant experiences. Companion-animal support was mentioned by only two participants and was not measured quantitatively. Multicenter and longitudinal studies are needed to assess the robustness and temporal ordering of these findings.

## 6 Conclusion

Family resources, perceived caregiving burden, family meaning-making, and patient sex were independently associated with total FHI score; other factors were associated with individual FHI subscales. Interviews situated these findings within caregivers’ accounts of obligation, changing roles, service access, resources, and coping. Companion-animal support was mentioned by two participants and remains hypothesis-generating. Prospective studies are needed to examine the temporal relationships among these factors and evaluate family-support interventions.

## Data Availability

The datasets presented in this article are not readily available due to the need to protect and respect the participants; they did not agree to data being shared beyond the objective of this study. Inquiries about the datasets can be directed to XL, lxf2015@lsu.edu.cn.
