# Supplementary material for: Family hardiness and associated factors among Chinese family caregivers of people with Alzheimer’s disease: an explanatory sequential mixed-methods study
Source: Front Psychol. 2026 Oct 14;17:1940356. doi: 10.3389/fpsyg.2026.1940356 (PMC13713748; doi:10.3389/fpsyg.2026.1940356)
Supplement: Supplementary file 1 [file Supplementary_file_1.docx]

**Supplementary table S1**

Measures of Candidate Factors Associated With Family Hardiness

| Influencing factors | Measures | Contents of measures | Cronbach’s α coefficient | |
| --- | --- | --- | --- | --- |
|  |  |  | Original | **Current** |
| 1. Stressors | | | | |
| 1.1 Activity of daily living | Chinese version Lawton-Brody Activity of Daily Living Scale (ADL-C) (Lawton and Brody, 1969; Zhang, 1996) | This 20-item caregiver-reported scale includes 8 basic ADL items (e.g., toileting, feeding, dressing) and 12 instrumental ADL items (e.g., telephone use, shopping, housekeeping). Items are rated on a 4-point Likert scale (1 = independent to 4 = completely dependent), yielding total scores from 20 to 80, with higher scores indicating greater functional impairment. | - | 0.983 |
| 1.2 Behavioral symptoms | Chinese version of Teri's (1992) Memory and Behavior Problems Checklist (RMBPC) (Teri,1992; Roth et al., 2003; Wang, 2018) | This 24-item caregiver-reported instrument assesses dementia-related behavioral symptoms using dichotomous scoring (0 = absent, 1 = present). Total scores range from 0 to 24, with higher scores indicating more frequent behavioral problems. | 0.870 | 0.900 |
| 2. Family coherence | Chinese version Family Coping Coherence Index (FCCI-C) (McCubbin, Thompson and McCubbin, 1996; Wang, 2018) | A 3-item caregiver-reported scale rated on a 5-point Likert scale (1 = strongly disagree to 5 = strongly agree). Total scores range from 3 to 15, with higher scores reflecting a more positive family outlook and stronger coherence. | 0.817 | 0.692 |
| 3. Family schema | Chinese version (Family Schema-Ethnic Index, FSCH-E-C) (McCubbin, Thompson and McCubbin, 1996; Wang, 2018) | A 30-item caregiver-reported measure assessing culturally embedded beliefs, values, spirituality, and family expectations. Items are rated on a modified 4-point Likert scale (0 = completely inconsistent to 3 = completely consistent), with an additional “-1 = never considered” option. Total scores range from -30 to 90, with higher scores indicating stronger cultural alignment and adaptive capacity. | 0.896 | 0.898 |
| 4. Family paradigms | Family Paradigms Scale (Wang, 2018) | A 33-item caregiver-reported instrument rated on a 5-point Likert scale (0 = not considered to 4 = consistently present). Total scores range from 0 to 132, with higher scores indicating stronger and more effective family caregiving paradigms. | 0.809 | 0.838 |
| 5. Family meanings | Chinese Positive Aspects of Caregiving Scale (Tarlow et al., 2004; Zhang, 2007) | This nine-item caregiver-reported scale assesses self-affirmation (items 1–4) and outlook on life (items 5–9). Items are rated from 1 (strongly disagree) to 5 (strongly agree), yielding a total score of 9–45. “Do not know” (coded −3) and “refuse to answer” (coded −4) are non-substantive responses, were treated as missing, and were not included in the total score. Higher scores indicate more positive meaning-making. | 0.900 | 0.903 |
| 6. Perceived caregiving burden | Chinese version of Zarit Burden Interview (ZBI-C) (Wang et al., 2006) | A 22-item caregiver-reported instrument assessing perceived caregiving burden across two dimensions: Personal Burden (items 1-12) and Responsibility Burden (items 13-22). Items are rated on a 5-point Likert scale (0 = never to 4 = always). Total scores range from 0 to 88, with higher scores indicating greater perceived burden. | 0.927 | 0.946 |
| 7. Family resources | Chinese version Family Inventory of Resources for Management (FIRM-C) (Wang et al., 2018) | A 51-item caregiver-reported scale assessing family resources across three dimensions: Family Control & Health Status (items 1-18), Economic Capacity (items 19-32), and Dignity & Communication (items 33-51). Items are rated on a 4-point Likert scale (0 = never to 3 = always). Total scores range from 0 to 153, with higher scores indicating greater resource availability. | 0.906 | 0.927 |
| 8. Family support system | | | | |
| 8.1 Family support | Chinese version Multidimensional Scale of Perceived Social Support (PSSS-C) (Jiang, 2001; Wang et al., 2018) | This caregiver-reported scale uses a 7-point Likert format (1 = extremely disagree to 7 = extremely agree), with total scores ranging from 4 to 28. Higher scores indicate stronger perceived family support. | 0.874 | 0.882 |
| 8.2 Kin support | Four items from (FIRM-C) (Wang et al., 2018) | Four-item kinship support subscale adapted from the FIRM-C. Items are rated on a 4-point Likert scale (0 = never to 3 = always), yielding total scores from 0 to 12. Higher scores reflect stronger kinship support. | 0.661 | 0.613 |
| 8.3 Community support | Chinese version Perceived Social Support Scale (Jiang, 2001) | This 8-item scale assesses support from significant others (e.g., neighbors, colleagues). Items are rated on a 7-point Likert scale, with total scores ranging from 8 to 56. Higher scores indicate stronger community support. | 0.831 | 0.933 |
| 8.4 Social support | Family Social Support Scale (Wang et al., 2018) | A 6-item caregiver-reported measure rated on a 7-point Likert scale, with total scores ranging from 6 to 42. Higher scores indicate stronger perceived social support. | 0.908 | 0.899 |
| 9. Problem solving and coping | Chinese version Family Crisis-Oriented Personal Evaluation Scales (F-COPES-C) (McCubbin, Thompson and McCubbin, 1996; Wang, 2018) | A 27-item caregiver-reported instrument assessing coping strategies across five dimensions: resource acquisition, social support seeking, positive reframing, spiritual coping, and avoidance (reverse-scored). Items are rated on a 5-point Likert scale (1 = strongly disagree to 5 = strongly agree). Total scores range from 27 to 135, with higher scores indicating more adaptive coping. | 0.842 | 0.843 |

Note: Teri, L., Truax, P., Logsdon, R., et al, 1992. Assessment of behavioral problems in dementia: the revised memory and behavior problems checklist. Psychol Aging 7 (4), 622–631.

Ngai, F.W., Ngu, S.F., 2011. Translation and validation of a Chinese version of the family sense of coherence scale in Chinese childbearing families. Nurs Res 60 (5), 295–301.

Wang, Q., 2018. The Verification of the Family Resilience Model under Chinese Cultural in the Family Care of Patients with Dementia. In: Department of Nursing. Peking Union Medical College, Beijing, China.

Zhang, R., 2007. A Study on the Caregiving Experience of Dementia Caregivers. In: Department of Nursing. Peking Union Medical College, Beijng.

Tarlow, B.J., Wisniewski, S.R., Belle, S.H., et al, 2004. Positive Aspects of Caregiving: Contributions of the REACH Project to the Development of New Measures for Alzheimer's Caregiving. Research on Aging 26 (4), 429–453.

Wang, L., Yang, X., Hou, Z., Feng, Q., Wang, J., 2006. Application and evaluation of chinese version of zarit caregiver burden interview. Chinese Journal of Public Health 22 (8), 970–972.

Roth, D.L., Burgio, L.D., Gitlin, L.N., et al, 2003. Psychometric analysis of the Revised Memory and Behavior Problems Checklist: factor structure of occurrence and reaction ratings. Psychol Aging 18 (4), 906–915.

Jiang, Q.J., 2001. Perceived Social Support Scale. Chinese Journal of Behavioral Medical Science 10 (10), 41–43.

Lawton, M.P., Brody, E.M., 1969. Assessment of older people: self-maintaining and instrumental activities of daily living. Gerontologist 9 (3), 179–186.

Zhang, M., 1996. The Application of Scales in the Diagnosis of Dementia. Chinese Journal of Practical Internal Medicine 13 (6), 337–339.

McCubbin, H.I., Thompson, A.I., McCubbin, M., 1996. Family measures: Stress, coping, and resiliency: Inventories for research and practice. University of Wisconsin.

**Supplementary table S2**

Demographic Characteristics of Caregivers and Family Profiles (N=201)

| **Characteristics** | **n (%) / Median (IQR)** |
| --- | --- |
| **Age** |  |
| <60 years | 93 (46.3) |
| Sex | 108 (53.7) |
| **Gender** |  |
| Male | 75 (37.3) |
| Female | 126 (62.7) |
| **Relationship to Patient** |  |
| Child | 103 (51.2) |
| Spouse | 75 (37.3) |
| Others | 23 (11.5) |
| **Education Level** |  |
| Primary school or below | 83 (41.3) |
| Junior high school | 48 (23.9) |
| Senior high/vocational | 34 (16.9) |
| College or above | 36 (17.9) |
| **Self-rated Health** |  |
| Good | 136 (67.7) |
| Fair | 53 (26.3) |
| Poor | 12 (6.0) |
| **Employment Status** |  |
| Employed | 77 (38.3) |
| Retired | 93 (46.3) |
| Unemployed | 31 (15.4) |
| **Monthly Household Income (RMB)** |  |
| <3000 | 25 (12.4) |
| 3000-6000 | 106 (52.7) |
| 6001-9000 | 52 (25.9) |
| >9000 | 18 (9.0) |
| **Number of Children** | 2 (2, 3)* |

*Median (25th percentile, 75th percentile)

**Supplementary table S3**

Coding of Independent Variables Examined in Relation to Family Hardiness Among Family Caregivers of People With Alzheimer's Disease

| **Variable** | **Assignment** |
| --- | --- |
| Patient sex | Male=0, Female=1 |
| Residence | Urban=0, Rural=1 |
| Education level | Primary/below=0 (ref), Junior high=1, Senior high/vocational=2, College+=3 |
| Caregiver health status | Good=0 (ref), Fair=1, Poor=2 |
| Caregiver occupation | Employed=0 (ref), Retired=1, Unemployed=2 |
| Continuous variables | Original values (e.g., family resources, social support, problem-solving) |

**Supplementary table S4**

Stepwise Multivariable Linear Regression for the Control Dimension of Family Hardiness (N = 201)

| Variables | *β* | *P* |
| --- | --- | --- |
| Patient residency | -0.348 | 0.004 |
| Perceived caregiving burden | -0.364 | <0.001 |
| Family meanings | 0.202 | 0.002 |
| *Note. R*^2^ = 0.268, adjusted *R*^2^ = 0.256, *F* = 23.99，*P* < 0.001, DW = 2.042，*P* = 0.786, χ² = 0.759，*P* = 0.859, MAX-VIF = 1.1, AD = Alzheimer's disease | | |

**Supplementary table S5**

Stepwise Multivariable Linear Regression for the Commitment/Responsibility Dimension of Family Hardiness (N = 201)

| Variables | *β* | *P* |
| --- | --- | --- |
| Problem solving and coping | 0.289 | <0.001 |
| Female patient sex | -0.249 | 0.020 |
| Perceived caregiving burden | -0.236 | <0.001 |
| Family paradigm | 0.220 | <0.001 |
| Family meanings | 0.163 | 0.019 |
| *Note: R*^2^ = 0.417, adjusted *R*^2^ = 0.402, *F* = 27.88，*P* < 0.001, DW = 1.974，*P* = 0.848, χ² = 0.922，*P*=0.969, MAX-VIF = 1.59, AD = Alzheimer's disease | | |

**Supplementary table S6**

Stepwise Multivariable Linear Regression for the Challenge Dimension of Family Hardiness (N = 201)

| Variables | *β* | *P* |
| --- | --- | --- |
| Family resources | 0.475 | <0.001 |
| Perceived caregiving burden | -0.179 | 0.004 |
| *Note. R*^2^ = 0.343, adjusted *R*^2^ = 0.336, *F* = 51.58，*P* < 0.001, DW = 1.851，*P* = 0.280, χ² = 1.296，*P* = 0.523, MAX-VIF = 1.210, AD = Alzheimer's disease | | |

**Supplementary table S7**

General Information of AD Patients and Their Family Caregivers (N=12)

| **ID** | **Patient Demographics** | | | | **Primary Caregiver Demographics** | | | | | |
| --- | --- | --- | --- | --- | --- | --- | --- | --- | --- | --- |
|  | Sex | Age  (years) | Duration of Illness (years) | Marital Status | Sex | Age (years) | Relationship to Patient | Education Level | Care Duration (years) | Caregiver's Occupation |
| P1 | Female | 87 | 9 | Married | Male | 57 | Son | College and above | 2 | Self-employed |
| P2 | Female | 78 | 0.5 | Unmarried | Male | 49 | Son | Technical secondary school/High school | 0.5 | Self-employed |
| P3 | Female | 90 | 5 | Married | Male | 90 | Spouse | College and above | 5 | Retired |
| P4 | Female | 70 | 15 | Married | Male | 68 | Spouse | College and above | 15 | Retired |
| P5 | Female | 87 | 3 | Unmarried | Female | 57 | Daughter-in-law | Junior high school | 1.8 | Unemployed |
| P6 | Female | 81 | 1 | Married | Male | 59 | Son | Technical secondary school/High school | 1 | Unemployed |
| P7 | Male | 81 | 4 | Married | Female | 78 | Spouse | Primary school and below | 4 | Unemployed |
| P8 | Male | 76 | 10 | Married | Female | 76 | Spouse | College and above | 10 | Retired |
| P9 | Female | 86 | 4 | Married | Female | 41 | Daughter-in-law | Primary school and below | 2 | Self-employed |
| P10 | Female | 76 | 6 | Unmarried | Male | 55 | Son | College and above | 6 | Self-employed |
| P11 | Male | 84 | 3 | Married | Female | 81 | Spouse | Junior high school | 3 | Retired |
| P12 | Male | 94 | 4 | Unmarried | Male | 69 | Son | Technical secondary school/High school | 4 | Self-employed |

**Supplementary File 1**

**Interview Guide**

This study developed the interview guide based on a theoretical model of family resilience in the Chinese cultural context (Wang, Q., 2018), findings from the cross-sectional survey in Part One, and expert consultations. Prior to formal data collection, a pilot interview was conducted with three primary caregivers of AD patients who met the inclusion criteria. The guide was subsequently revised based on their feedback and finalized for use in the formal interviews.

The formal interview guide is as follows:

1. Could you describe your family—who lives in the household, and what a typical day has been like since you began caring for the person with AD?
2. How is the patient’s health currently? When did your family first notice that something was wrong, and how did you come to realize it?
3. What went through your mind and your family’s minds when you learned about the diagnosis? How did everyone react? And what did this situation come to mean for your family as time went on?
4. What roles does each family member play within the household? Are there any unspoken rules or agreements that your family tends to follow? In what ways have these roles and rules been helpful — or not — as your family has navigated this difficult time?
5. How would you and your family rate the level of stress you are currently under? What are the main sources of that stress?
6. What resources does your family draw on when facing these pressures? And how has this caregiving affected your own and your family’s health — physically and emotionally?
7. How have you and your family coped with and adapted to these stresses over time?
8. Has your family gained any new insights or perspectives through this experience? If so, how might those insights shape the way your family moves forward?
